# A next-generation marker genotyping platform (AmpSeq) in heterozygous crops: a case study for marker-assisted selection in grapevine

**DOI:** 10.1038/hortres.2016.2

**Published:** 2016-02-17

**Authors:** Shanshan Yang, Jonathan Fresnedo-Ramírez, Minghui Wang, Linda Cote, Peter Schweitzer, Paola Barba, Elizabeth M Takacs, Matthew Clark, James Luby, David C Manns, Gavin Sacks, Anna Katharine Mansfield, Jason Londo, Anne Fennell, David Gadoury, Bruce Reisch, Lance Cadle-Davidson, Qi Sun

**Affiliations:** 1 Horticulture Section, School of Integrative Plant Science, Cornell University, Geneva, NY 14456, USA; 2 Bioinformatics Facility, Cornell University, Ithaca, NY 14853, USA; 3 Institute of Biotechnology, Cornell University, Ithaca, NY 14853, USA; 4 Plant Breeding and Genetics Section, School of Integrative Plant Science, Cornell University, Ithaca, NY 14853, USA; 5 Department of Horticultural Science, University of Minnesota, St Paul, MN 55108, USA; 6 Department of Food Science, Cornell University, Geneva, NY 14456, USA; 7 Department of Food Science, Cornell University, Ithaca, NY 14853, USA; 8 USDA-ARS Grape Genetics Research Unit, Geneva, NY 14456, USA; 9 Plant Science Department, South Dakota State University, Brookings, SD 57007, USA; 10 Plant Pathology and Plant-Microbe Biology Section, School of Integrative Plant Science, Cornell University, Geneva, NY 14456, USA

## Abstract

Marker-assisted selection (MAS) is often employed in crop breeding programs to accelerate and enhance cultivar development, via selection during the juvenile phase and parental selection prior to crossing. Next-generation sequencing and its derivative technologies have been used for genome-wide molecular marker discovery. To bridge the gap between marker development and MAS implementation, this study developed a novel practical strategy with a semi-automated pipeline that incorporates trait-associated single nucleotide polymorphism marker discovery, low-cost genotyping through amplicon sequencing (AmpSeq) and decision making. The results document the development of a MAS package derived from genotyping-by-sequencing using three traits (flower sex, disease resistance and acylated anthocyanins) in grapevine breeding. The vast majority of sequence reads (⩾99%) were from the targeted regions. Across 380 individuals and up to 31 amplicons sequenced in each lane of MiSeq data, most amplicons (83 to 87%) had <10% missing data, and read depth had a median of 220–244×. Several strengths of the AmpSeq platform that make this approach of broad interest in diverse crop species include accuracy, flexibility, speed, high-throughput, low-cost and easily automated analysis.

## Introduction

Marker-assisted selection (MAS) is now commonly employed in perennial crop breeding programs to pursue the acceleration of cultivar development.^1–3^ In particular, MAS has been shown to provide advantages for selection during the juvenile phase;^4,5^ for pyramiding disease resistance genes;^6,7^ and for replacing expensive, time-consuming or technically difficult traits.^8,9^ Simply inherited traits with Mendelian or near-Mendelian segregation patterns are major targets for MAS. Examples of MAS have been reported for seedlessness and flower sex in grape, and disease resistance in apple, grape and tomato breeding.^1,10,11^ Markers have also been applied to quantitatively inherited traits, especially those with major quantitative trait loci (QTL) effect, including fruit acidity in peach,^12^ fruit size in tomato,^13^ peach and cherry,^14^ grain yield in rice^15^ and drought tolerance in chickpea.^9^

The development of molecular markers requires the detection of association between target traits and genotypes. Two approaches are often used to detect such associations: (a) QTL analysis with structured families, and (b) genome-wide association study, which takes advantage of linkage disequilibrium (LD) in diverse germplasm to capture the linkage between markers and causal genes.^16,17^ However, for highly heterozygous and diverse crops, such as grape, genome-wide association study has limitations.^18–20^ LD decays rapidly in species of *Vitis*, in which the square of the correlation coefficient (*r*^*2*^) declines to 0.1 within 2.7 cM, making genome-wide association study an unsuitable method for genetic mapping with current genotyping platforms, such as single nucleotide polymorphism (SNP) microarrays.^21,22^ QTL analysis in mapping families has been a more effective method for genetic mapping in species with diverse backgrounds.^21^

Multiple genotyping platforms and molecular marker types have been utilized for MAS in highly heterozygous crops, including simple sequence repeats (SSR) and single-locus or multi-locus SNP assays. SSRs are particularly well-suited for MAS because of their multi-allelic nature and high transferability among distinct species or genera,^23–25^ which enables the analysis of complex crossing involving progenitors with multiple interspecific hybridizations. However, SSR as a genotyping platform has its own disadvantages including low-throughput, labor-intensive and time-consuming.^26^ Low density of SSR markers could cause loss of linkage between markers and causal genes, or lack of segregation in certain families.^27,28^ SNP microarrays emerged as an alternative high-throughput genotyping platform.^29^ Commercially available high density oligonucleotide arrays allow parallel genotyping for thousands of individuals or markers.^30^ However, SNP microarrays are closed platforms suffering from ascertainment bias,^31^ resulting in poor flexibility and poor transferability across diverse germplasm.^32^ In addition, the cost of microarray pre-design is still a major obstacle in adopting SNP arrays in horticultural breeding programs.^33,34^

To fill the gaps between the above technical and economic considerations for breeding and actual implementation of MAS,^35^ next-generation sequencing (NGS) technology offers a potential opportunity for unbiased genotyping with high-throughput and low per-sample cost. Genotyping-by-sequencing (GBS), with its simultaneous marker discovery and genotyping approach, delivers many benefits including availability of flanking DNA sequence information, high-sample throughput and scalability (multiplexing), and high resolution.^36^ It has been successfully applied in marker discovery for many self-pollinating crops as well as outcrossing species.^37^ However, successful implementation of GBS for MAS has not been reported in any heterozygous crop breeding programs. Many reasons may have contributed to the lack of adoptions. From a technical perspective, missing data, genotyping errors and heterozygote under-calling are common in GBS results due to uneven sequencing depth across sites and high level of sample multiplexing.^38–41^ Rapid LD decay, large-scale genome structure variation coupled with lack of haplotype information makes it impractical to do genotype imputation in heterozygous perennial species with diverse backgrounds. From a practical perspective, the long turn-around time from sample collection to data analysis makes it difficult to fit into most breeding timeframes. Moreover, computational challenges in data processing further hinder breeders’ interest in implementing GBS. In summary, GBS is currently impractical for MAS in heterozygous crops.

This study presents a novel and efficient strategy for molecular marker development and practical implementation in MAS, based on amplicon sequencing (AmpSeq). The semi-automated pipeline incorporates a machine learning model for primer design and uses Illumina’s Nextera dual-barcoding and sequencing platforms for genotyping (Illumina, San Diego, CA, USA). After detecting a SNP from GBS, the strategy starts from the design of primers using the GBS sequence tags. The converted amplicon markers can then be used for genotyping through NGS. The design involves multiplexing of both samples and markers. As a case study, we document the use of AmpSeq in grapevine breeding programs for three traits including flower sex, powdery mildew (PM) resistance and acylated anthocyanins. We chose these three traits to represent a Mendelian trait and two QTL with differing effects on phenotypic variance: (a) a single gene for flower sex with three alleles^42^; (b) a QTL with moderately high *R*^2^ (acylation of anthocyanins, initially reported here); and (c) a QTL with relatively low *R*^2^ (*Ren2* locus for PM (*Erisyphe necator*) resistance from *Vitis cinerea* (Engelm. ex A. Gray) Engelm. ex Millard accession B9 (*V. cinerea* B9)^43,44^). All three loci are located on different chromosomes, and we were able to test the AmpSeq approach for flower sex (male versus female versus hermaphrodite) across four different families where the male flower allele descends from *V. cinerea*, the hermaphrodite flower allele descends from *V. vinifera* L. and the female allele descends from an unidentified North American *Vitis* species. Two of the three traits chosen for analysis would take 2–4 years to analyze phenotypically due to the time it takes for a seedling to produce flowers and fruit. We also report here the development of a pipeline package with tools for AmpSeq marker design and decision support.

## Materials and methods

### Plant materials

Four families were chosen for this study, all representing interspecific hybridization of diploid (2*n*=38) *Vitis* species: *V. vinifera* ‘Chardonnay’×*V. cinerea* B9; ‘Horizon’ (complex hybrid of *V. vinifera*, *V. labrusca* L., *V. rupestris* Scheele and *V. aestivalis* Michx.)×*V. cinerea* B9; ‘Horizon’×Illinois 547-1 (*V. rupestris* B38×*V. cinerea* B9) and MN1246×MN1264 (both, complex hybrids with a genomic background including at least *V. vinifera, V. riparia* Michx.*, V. rupestris, V. labrusca, V. cinerea* and *V. aestivalis*). The first three populations were grown in research vineyards operated by Cornell University, Geneva, New York. The latter population was grown in research vineyards of the University of Minnesota, St Paul, located at Excelsior, Minnesota. All four populations segregated for flower sex (male, female and hermaphrodite) a trait controlled by a single locus with three alleles (reviewed in the study by Hyma *et al.*^42^) In addition, our ongoing research indicated that two populations segregated for acylation of anthocyanins, and the three populations descending from *V. cinerea* B9 segregated for *Ren2* PM resistance.^43,44^

### AmpSeq marker development pipeline

**Figure 1:**
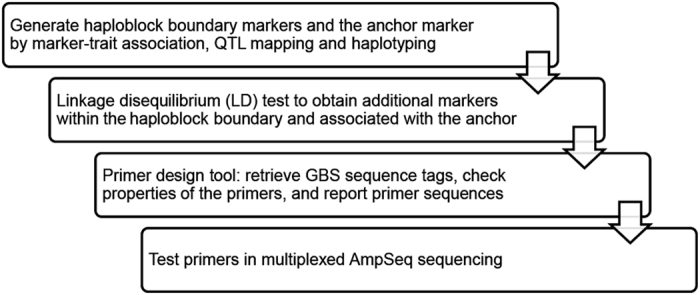
Workflow of the AmpSeq strategy.

The AmpSeq marker development procedure consists of four steps illustrated in Figure 1. First, GBS marker-trait associations were evaluated in TASSEL 4.3.13 (ref. 45). Genetic maps were constructed by the HetMappS strategy, and QTL were mapped in R/qtl ver. 1.37–11 as described by Hyma *et al.*^42^ Marker phase and effect were estimated by a custom R script (Supplementary File 1) using the intermediate files ‘phased’ and ‘LGmap’ from the HetMappS pipeline.^42^ For the second step, the range of the haploblock across the QTL region was defined by haploblock start and end markers with similar absolute value of marker effects. The anchor marker selected for each QTL was based on high association with trait within the 1.8-LOD (logarithm base 10 of odds) support interval of the QTL. A Perl script included in the package ‘calculated_LD_distribution.pl’ took in a text file ‘test_marker’ as input, which includes physical positions of the three markers: haploblock start, haploblock end and the anchor SNP. This script retrieved additional SNP markers from the GBS pipeline (pre-filtered by maximum of 5% missing data) that were within the haploblock range and verified to be in LD with the anchor marker.

After the SNP markers were defined, a Perl script ‘parse_bam.pl’ was used to identify genomic regions suitable for designing PCR primers. The template for the primer sequences was based on the actual sequences from GBS data, rather than the reference genome sequence itself. This was to ensure that there was no mismatch between the primer sequences and their targeted alleles. The ‘parse_bam.pl’ script required three inputs: (1) the reference genome FASTA file; (2) a BAM file, converted from the SAM file generated by TASSEL GBS pipeline,^45^ which contained GBS reads aligned to the reference genome; and (3) a cutoff for the LD *P*-value as calculated from the previous step. By default, the script required that at least 10 bp of the left and right flanking sequences are covered by the GBS read, and ⩾90% alignment matching rate between the two alleles of up to 20 bp of each flanking sequences. Finally the script ‘primer3.pl’ was used to call the primer designing software Primer3 (ref. 46) and captured output properties from Primer3. The default primer sequence length from the pipeline was 22 bp. With a single SNP in the middle, the targeted amplicon size is 45 bp. From the output of the primer design pipeline followed by manual filtering, including removal of overlapping amplicons and primers with extreme annealing temperatures (*T*_m_) outside of 47–79 °C, a total of 54 amplicons across the three loci were retained for testing: 19 for flower sex, 12 for PM resistance and 23 for acylated anthocyanins. The output file of the 54 amplicons is shown in Supplementary File 2. The documentation about the usage of the pipeline and the scripts are provided in Supplementary File 3.

### Genotyping (PCR, library preparation and de-multiplex)

Two experiments were conducted to test three traits: flower sex, PM resistance due to the *Ren2* locus and acylated-anthocyanin concentration. In Experiment 1, 19 primers for flower sex and 12 primers for *Ren2* were pooled in one Illumina MiSeq lane (Illumina, San Diego, CA, USA) testing four 96-well plates (380 individuals and 4 blanks), each containing the parents and a subsample of progeny from each grapevine breeding family. Experiment 1 consisted of four breeding families: ‘Horizon’×*V. cinerea* B9, ‘Horizon’×Illinois 547-1, and ‘Chardonnay’×*V. cinerea* B9 and MN1246×MN1264. The other 23 primers for acylated-anthocyanin concentration were pooled in Experiment 2, testing two 96-well plates of ‘Horizon’×Illinois 547-1 and two 96-well plates of *V. rupestris* B38×‘Horizon’ (380 individuals and 4 blanks).

For each vine, a single small leaf (<1-cm diameter) was harvested and placed in one tube of a Costar 96-well cluster tube collection plate (Corning, Corning NY, USA), and DNA was isolated as described previously.^42^ Briefly, each 96-well plate received up to 91 unique samples plus two sets of duplicated individuals and a blank well to serve as quality controls. Frozen samples were ground using stainless-steel beads in a Geno/Grinder 2000 (OPS Diagnostics LLC, Lebanon NJ, USA) and processed using DNeasy 96-well DNA extraction kits (Qiagen, Valencia CA, USA). The Qiagen AP1 lysis buffer was amended with PVP-40 (2% w/v) prior to heating of the buffer, to improve DNA quality and quantity. Due to historically consistent yields of 25–50 ng μl^−1^ within and between plates, DNA was used following twofold dilution without quantification.

AmpSeq uses two rounds of PCR: the 1st PCR to amplify a multiplex of markers and incorporate linker sequences, and the 2nd PCR to use the linker sequences to add a unique pair of indices, or barcodes, to each sample. The linker 5′-TCGTCGGCAGCGTCAGATGTGTATAAGAGACAG-3′ was added to the 5′ end of each forward primer to accommodate S5xx barcode adapters, and the linker 5′-GTCTCGTGGGCTCGGAGATGTGTATAAGAGACAG-3′ was added to the 5′ end of each reverse primer to accommodate N7xx barcode adapters described in Supplementary File 4. For results presented here, samples were processed without previously testing the primers as a multiplex, and both rounds of PCR were conducted on 384 wells per batch, processed as four 96-well plates, which were pooled after the 2nd PCR for Illumina sequencing.

For the 1st PCR, up to 31 primer pairs (62 total, 10 pmol each) were combined with Qiagen Multiplex PCR Plus Mix (Qiagen, Valencia CA, USA), following the manufacturer’s protocols calculated for 10 μl reaction volumes including 2 μl of DNA template. Thermocycling conditions for the 1st PCR were: 95 °C for 5 min; followed by 35 cycles of 95 °C for 30 s, 62 °C for 90 s and 72 °C for 30 s; followed by a final extension of 10 min at 68 °C. After the 1st PCR, 50 μl H_2_O was added to each well prior to serving as a template for the 2nd PCR.

In the 2nd PCR, 384 unique combinations of 16 row and 24 column indices were incorporated in 10 μl reactions consisting of Platinum Taq (2 U per reaction, Invitrogen, Grand Island NY, USA), Platinum Taq buffer (1× final), MgCl_2_ (1.5 mM final), dNTPs (0.2 mM final each), primers (10 pmol each) and 2 μl of each amplicon pool from the 1st PCR reaction. Thermocycling conditions for the 2nd PCR were: 95 °C for 5 min; followed by eight cycles of 95 °C for 30 s, 53 °C for 30 s and 72 °C for 30 s; followed by a final extension of 10 min at 68 °C. Equal volumes of all 384 wells were pooled into a single tube, then purified by Ampure (Beckman Coulter, Indianapolis IN, USA), following manufacturer’s instructions.

The amplicon pool was sequenced on an Illumina MiSeq instrument with single-end reads of 51 bp with 8 bp, dual index reads according to the manufacturer’s instructions. Reads were de-multiplexed using the Illumina bcl2fastq pipeline software, allowing a single mismatch in the index reads.

### Phenotyping and data analysis

The experimental design for phenotyping is described in Supplementary File 5 (refs 42,47,48,49).

SNPs were called by a custom perl script ‘run_gatk2.pl’ (Supplementary File 6) from the de-multiplexed fastq file. Read counts were calculated in 500 bp sliding windows across the 12X.2 version of the PN40024 reference genome^50^ using BEDTools^51^ v2.22.1 to check the amplification specificity and efficiency of the AmqSeq markers. VCFtools^52^ v0.1.12a was applied to calculate missing rate per site (--missing-site) and per individual (--missing-indv), and mean depth per site (--site-mean-depth) and per individual (--depth). VCF file generated from ‘run_gatk2.pl’ was input to TASSEL 4.3.13 for association detection. Genotyping and phenotyping data of the two parents were used for phasing.

To detect family-based marker specificity, the squared correlation coefficients between AmpSeq markers were calculated per family for each trait by VCFtools--geno-r2 function, following a dendrogram construction in R^53^ v3.1.2. *P*-values of association between genotype and phenotype were calculated by a one-way analysis of variance co-segregation analysis General Linear Model (GLM) on a per-family basis and after pooling families with the same segregation pattern. The minor allele frequencies of the AmpSeq markers were also reported on a per-family and pooled basis.

Output from the primer design pipeline was used to classify primers as efficient and non-efficient based on their amplification and genotyping performance, and 4 statistical models (logistic regression, support vector machine, decision tree and random forest) were tested using the following cross-validation method for assessing 18 numerical parameters of each primer (num_tags, pvalue, phase, reject_code, a1, a1_count, a1_lseq_pct, a1_rseq_pct, a2, a2_count, a2_lseq_pct, a2_rseq_pct, ltemp, rtemp, a1_lseq_len, a1_rseq_len, a2_lseq_len, a2_rseq_len). In the cross-validation algorithm, the efficient and non-efficient categories are randomly partitioned into two groups, the training group and the testing group, and the testing group is used for testing the performance of four statistical models trained with the training group. The prediction accuracy of the cross-validation classification model was assessed using Receiver Operating Characteristic (ROC) curve analysis.^54–57^ The ROC curve is a commonly used diagnostic to assess the prediction power of different classification methods, by means of a graphical plot of the true positive rate (sensitivity) against the false positive rate (1—specificity) for each classification method that predicts a dichotomous outcome. The plot is showed at several thresholds, which are used to designate whether the prediction of a given method as positive. The area under this curve (AUC) is one of the most important performance metrics that can be applied for selecting the most adequate classification method because it represents the accuracy of the prediction. In practice, AUC values range from 0.50 to 1.00, and a higher AUC value indicates better prediction accuracy for the classification models: values below 0.60 should be considered poor, 0.60–0.74 are moderate, 0.75–0.89 are good and ⩾0.90 are very good to excellent.^57^ A value of exactly 0.50 would indicate that the model is useless for testing, while a value of exactly 1.00 would indicate the model is a perfect test. The models were evaluated using packages caret, e1071, rpart and random forest implemented in R v3.1.2.

## Results

### Converting GBS-derived SNPs to amplicon sequencing (AmpSeq) markers

The procedure for the marker development and amplicon sequencing primer design pipeline is illustrated in Figure 1. Three traits were chosen for study: flower sex, *Ren2* PM resistance^43,44^ and acylated anthocyanins (that is, anthocyanins esterified to hydroxycinnamic acids). The percentage of phenotypic variance explained by each QTL (*R*^*2*^ value) confirmed these represent a qualitative trait (93%), a quantitative trait with a moderate QTL (13%), and a quantitative trait with a major QTL (54%), respectively (Table 1). For each locus, two flanking markers indicating the haploblock boundary, and one anchor marker having high association with the phenotype from the curated genetic maps are listed in Table 1. The primer design pipeline and curation resulted in 54 AmpSeq markers physically located between the two flanking markers and genetically linked to the anchor markers, for further testing (Supplementary File 2).

In Experiment 1, 12,677,206 reads were generated for the target QTL region on chromosome 2 (chr2) associated with flower sex, and 4,537,611 reads were generated for the target QTL region on chr14 associated with *Ren2* PM resistance. These 17,214,817 reads at the two target loci comprise 99.99% of the total reads, while only 2,237 reads aligned to off-target regions. Similar results were obtained in Experiment 2, with 14,927,137 reads (98.82%) aligning to the target QTL region on chr 3 associated with acylated anthocyanins.

The sequencing depth per individual and missing rate were evaluated for each amplicon (Tables 2,3, 4). For both experiments, a majority of amplicons (80% in Experiment 1 and 83% in Experiment 2) had >50-fold coverage per individual, in a relatively narrow range from 50–284×. Most amplicons (87% in Experiment 1 and 83% in Experiment 2) had <10% missing data. Thus, in Experiment 1, the 380 individuals had 220× median coverage per marker and 168× mean coverage per marker. Similarly in Experiment 2 for 23 acylated-anthocyanin AmpSeq markers, 380 individuals had genotyping data with 244× median coverage per marker and 188× mean coverage per marker. One amplicon per experiment failed because no polymorphism was identified during the SNP calling procedure. The amplification specificity, stable sequencing depth and consistent missing rate in the two experiments indicated the marker development strategy and amplicon primer design pipeline were effective for all three loci, with no obvious bias during amplification, pooling or sequencing.

### Evaluation and validation of AmpSeq markers in grapevine breeding families

#### Flower sex

**Figure 2:**
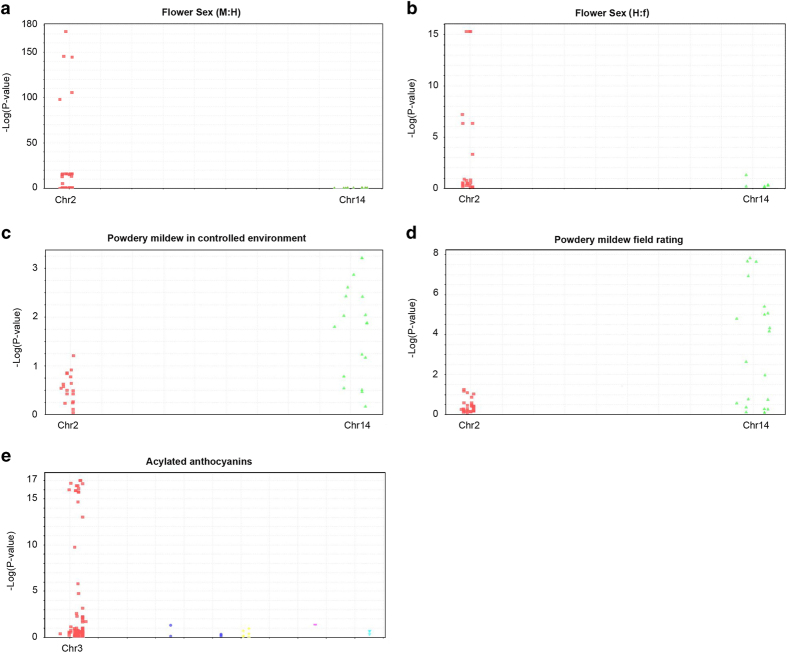
Manhattan plots of pooled association tests between AmpSeq markers and three traits. (**a**) Flower sex trait with male/hermaphrodite (M/H) segregation (‘Horizon’×Illinois 547-1, ‘Horizon’×*V. cinerea* B9 and ‘Chardonnay’×*V. cinerea* B9); (**b**) Flower sex trait with hermaphrodite/female (H/f) segregation (MN1246×MN1264); (**c**) Powdery mildew resistance assessed in a controlled environment (transformed mean of total hyphal transects *in vitro*) (‘Horizon’×*V. cinerea* B9); (**d**) Powdery mildew resistance assessed visually in the field (1–5 scale based on natural infection) (‘Horizon’×Illinois 547-1); (**e**) Acylated-anthocyanin content (‘Horizon’×Illinois 547-1 and *V. rupestris* B38×‘Horizon’).

To evaluate the 19 AmpSeq markers for flower sex, genotypic data were obtained for four breeding families in Experiment 1. This trait is reportedly controlled by a single locus with three alleles, with male (M) dominant over hermaphrodite (H), which is dominant over female (f).^42^ Three families segregated 1:1 for M/H—‘Horizon’×Illinois 547-1, ‘Horizon’×*V. cinerea* B9 and ‘Chardonnay’×*V. cinerea* B9. One family segregated 1:1 for H/f—MN1246×MN1264. Parents of the four families represent three phenotypes: ‘Horizon’ (HH), ‘Chardonnay’ (HH) and MN1264 (Hf) produce hermaphrodite flowers; Illinois 547-1 (Mf) and *V. cinerea* B9 (Mf) produce male flowers; and MN1246 (ff) produces female flowers.^42^ Amplicon data were merged with flower sex field ratings for a one-way analysis of variance co-segregation analysis. For the pooled association test using families with the same segregation pattern, only 1 amplicon had a non-significant association, and 16 out of 18 amplicons had a −log_10_(*P*-value) >13, suggesting a high association between AmpSeq markers and the recorded phenotypes (Figures 2a and b and Table 2). The high *R*^*2*^ value of each marker was consistent with the high *R*^*2*^ value of the flower sex QTL (93%, Table 1). Interpretation and direct application of AmpSeq genotypes for MAS required phase information, details of which are documented in Supplementary File 7.

**Figure 3:**
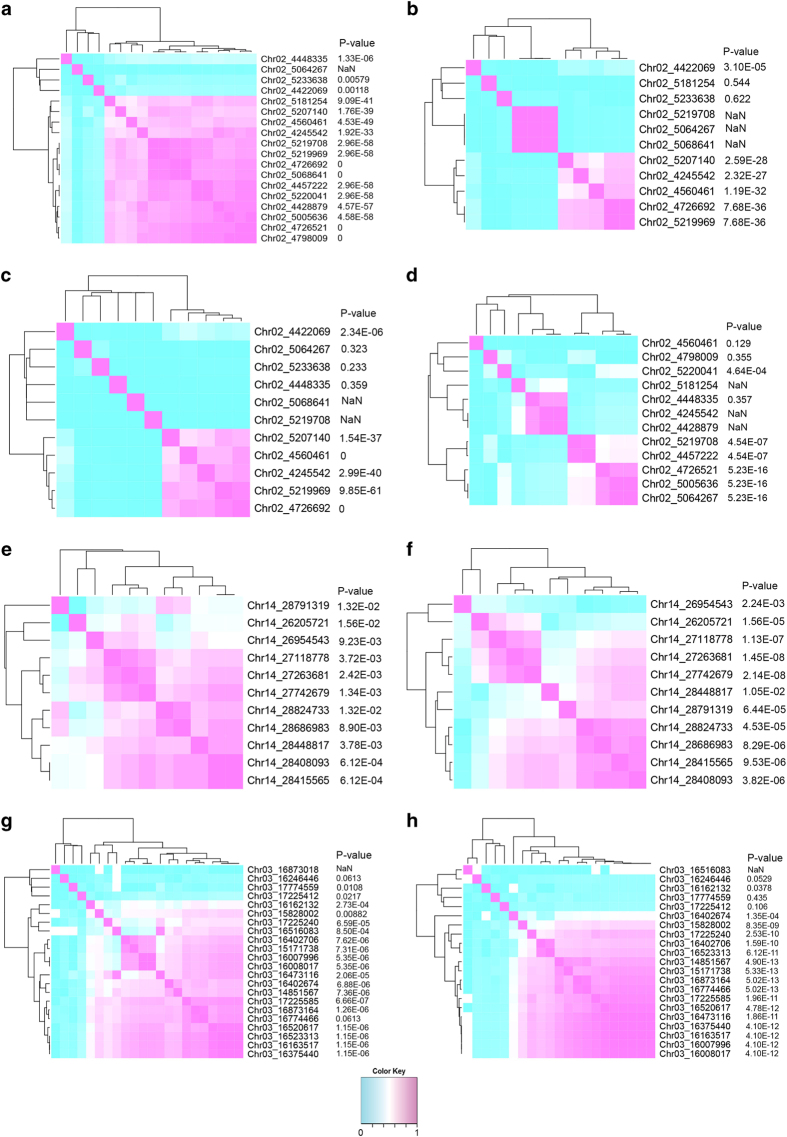
Marker transferability explored by dendrogram analysis with *P*-values of marker-trait association. (**a**) Flower sex for ‘Horizon’×Illinois 547-1; (**b**) Flower sex for ‘Horizon’×*V. cinerea* B9; (**c**) Flower sex for ‘Chardonnay’×*V. cinerea* B9; (**d**) Flower sex for MN1246×MN1264; (**e**) Powdery mildew resistance assessed in controlled environment for ‘Horizon’×*V. cinerea* B9; (**f**) Powdery mildew resistance assessed visually in the field for ‘Horizon’×Illinois 547-1; (**g**) Acylated-anthocyanin content for ‘Horizon’×Illinois 547-1; (**h**) Acylated-anthocyanin content for *V. rupestris* B38×‘Horizon’. Color key below indicates that shades of red color represent high correlation, while shades of blue color represent low correlation.

To shed light on marker transferability among breeding populations, genotype-phenotype association and minor allele frequency were reported by family in Table 3 and compared using a dendrogram (Figures 3a–d). AmpSeq markers with similar *P*-values and *R*^*2*^ patterns clustered together. Some AmpSeq markers in certain families have an *R*^*2*^ value equal to 1, suggesting the potential of 100% predictive accuracy in explaining 100% of the phenotypic variance.

#### PM resistance

To evaluate the 12 AmpSeq markers for *Ren2* PM resistance, genotypic data from Experiment 1 were merged with phenotypes from two families, each having a different phenotyping approach: Set 1 used 78 progeny of ‘Horizon’×*V. cinerea* B9 scored using the transformed mean of total hyphal transects *in vitro*; Set 2 used visual ratings (on a 1–5 scale) of natural infection in the vineyard for 91 progeny of ‘Horizon’×Illinois 547-1.

For Set 1, eight markers significantly predicted resistance [−log_10_(*P*-value) >2], and the marginal *P*-values and small *R*^*2*^ values (Figure 2c, Table 3) reflected the moderate QTL (*R*^*2*^=13%) discovered by GBS. For Set 2, 11 of 12 markers were significant (−log_10_(*P*-value) >2) (Figure 2d) with an average of 5.3, and all 12 markers explained more variance for vineyard ratings than for *in vitro* hyphal transects. The results confirmed that AmpSeq markers for *Ren2* PM resistance worked in a separate but related breeding family, even when evaluated by a different phenotyping method. A majority of the amplicons (9/12) can be used to track resistant alleles in phase, meaning that the minor allele is associated with PM resistance. AmpSeq markers with similar *P*-values and *R*^*2*^ patterns clustered together (Figures 3e and f).

#### Acylated anthocyanins

To evaluate the 22 AmpSeq markers for acylated-anthocyanin content, association tests were executed on 182 individuals including: 82 progeny from ‘Horizon’×Illinois 547-1, a subset of the original family for marker development, and 100 progeny from *V. rupestris* B38×‘Horizon’ to evaluate marker transferability. In a pooled analysis, 1 amplicon had a non-significant association, and 15 of 22 amplicons had a −log_10_(*P*-value) >10 (Figure 2e, Table 4). Individually, these 15 AmpSeq markers explained an average of 33% of the phenotypic variance, comparable to the QTL contributing 54% of the phenotypic variance. As with the flower sex dendrogram, AmpSeq markers with similar *P*-values and *R*^*2*^ patterns clustered together (Figures 3g and h).

### Criteria of efficient AmpSeq markers

**Figure 4:**
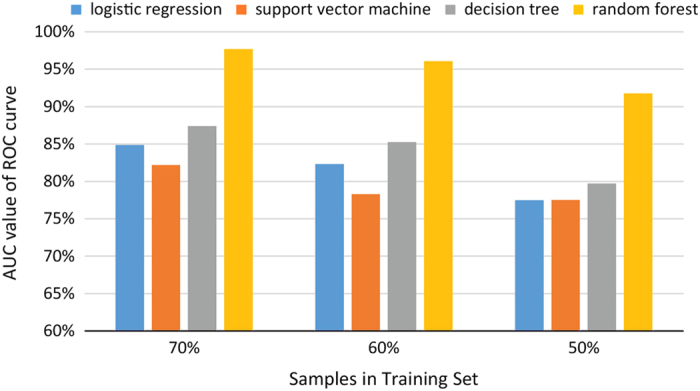
Evaluation of four machine-learning models for performance prediction of AmpSeq markers.

To reduce costs for primer synthesis and testing, we tested models to determine *post hoc* the relative importance of AmpSeq marker parameters to predict the most effective markers. Four statistical models (logistic regression, support vector machine, decision tree and random forest) were tested using quantified parameters of each primer to classify the primer output from the primer design pipeline into efficient and non-efficient categories. In all cases, regardless of the training set size, the random forest model (with over 90% predictive accuracy) outperformed the other three models (Figure 4). Thus, we developed a decision support tool based on the random forest model (Supplementary File 8), which can be used to facilitate AmpSeq marker selection following the primer design pipeline. The most important parameters in this model included the *P*-value of LD with the anchor marker, the primer annealing temperatures, the rejection code and the length of the sequence flanking each side of the SNP (Supplementary File 8).

## Discussion

This study had two main goals: (1) to develop a cost-effective and robust genotyping platform for MAS in heterozygous crops, and (2) to generate marker sets for MAS in grapevine breeding. A semi-automated primer design pipeline was developed to convert GBS tags to AmpSeq markers. The primer design procedure was facilitated by a decision support tool to predict which primers would perform appropriately in terms of predictability based on a pre-trained random forest model. For grapevine breeding, AmpSeq markers were developed for three economically important and representative traits with high breeding value: flower sex, PM resistance and acylated-anthocyanin concentration. The strategy was effective for all three traits given the diverse background and high heterozygosity of grapevine. In the process, 54 markers were tested on a total of 760 individuals in 6 breeding families involving 7 *Vitis* species across 2 breeding programs. The results indicated that the majority of AmpSeq markers have potential for accurate trait predictions, and a MAS package can be implemented for interspecific hybrid families.

### Technical considerations of the AmpSeq strategy

Two key decision points for the usage of the AmpSeq primer design pipeline were the selection of an anchor marker and the definition of haploblock boundaries of each QTL. The optimal parameters depended on the nature of the trait and mapping family, and here we describe what worked for these three traits. First, each anchor marker was selected from our final GBS linkage map, after the HetMappS pipeline and genetic map curation^42^ removed SNPs that had segregation bias, low genotyping rate, putative sequencing error, redundancy or aligned to repetitive genome regions. Thus, the anchor marker was selected from high-quality, mapped SNPs. Second, the anchor marker had a high *P*-value for marker-trait association. Third, the anchor marker was within the 1.8 LOD interval of the QTL to ensure tight genetic linkage. The anchor marker was used to retrieve SNPs in LD from an un-filtered VCF file within the haploblock defined by the two boundary markers, to increase marker density under the QTL region and provide more alternatives for AmpSeq marker design. The range of the haploblock can be defined by the marker effect as suggested in ‘Materials and methods’, or by other haplotyping software specific for heterozygous species, like iXora^58^ and SHAPEIT2 (ref. 59).

Two custom options can be set for the amplicon primer design pipeline: one is *P*-value threshold of the LD test, and the other one is the primer size, which was specified as 22 in this study to accommodate the 50 bp sequencing length and data analysis pipeline. To optimize the *P*-value of the LD test, all the parameters output from the pipeline were explored by four models (logistic regression, support vector machine, decision tree and random forest), in which the random forest model was the most useful to predict high-quality markers. In the random forest model, four primer parameters primarily contributed to the decision: the *P*-value of LD with the anchor marker, the rejection code and the annealing temperatures (*T*_m_) of the forward and reverse primers. Supported by the split value of the decision tree model (Supplementary File 9) and general guidelines for PCR based on experimental experience and thermocycling conditions used here, the recommended criteria for efficient amplicon markers includes: (1) the default *P*-value of LD with the anchor marker should be 1e−25; (2) *T*_m_ of primers should be between 52 and 70 °C to obtain high depth and avoid missing data; (3) the rejection code should be 0, representing exactly two alleles identified in GBS tags; (4) extremely high read depths of GBS tags, which may imply presence of a repetitive genome region, should be filtered; and (5) overlapping amplicons, which would result in cross-amplification, should be excluded. The pooling strategy employed in the study (Experiment 1: 384 individuals with 31 amplicon markers for 2 traits; Experiment 2: 384 individuals with 23 amplicon markers for 1 trait) resulted in read depths greater than 50×, low missing rate and equal amplification efficiency, suggesting that multiplexing could be increased for more traits, markers or individuals.

In terms of predictive power of converted markers, the *R*^*2*^ value of the QTL plays a major role. For a Mendelian trait such as flower sex, in which one major locus can explain 93% of phenotypic variance, the association between genotypes and phenotypes is highly significant. Some AmpSeq markers approached 100% predictive accuracy, as they can explain 100% of phenotypic variance in a given family. In contrast, for the major QTLs contributing <50% phenotypic variation, the predictive power is lower, which is consistent with theoretical framework.^60^ However, the predictive power is still attractive in breeding, when phenotypic screening is expensive and challenging. The notion was supported by several studies showing selection performed combining MAS with phenotypic evaluation is more efficient than selection based on phenotyping alone, especially when the family is large and trait heritability is low.^8,61–65^ Minor allele frequencies were calculated of each AmpSeq marker for each segregating family (Table 2). Segregation distortion, defined as observed minor allele frequency deviating from expected minor allele frequency of 0.25, was observed for some AmpSeq markers in all analyses. Similar distortion has been reported for SSR markers in grape,^43^ for GBS markers in rice^15^ and for sequence-tagged microsatellite site in chickpea.^66^ Although simulations claim that the presence of marker segregation distortion has little effect on linkage map construction and QTL analysis,^67,68^ distortion has resulted in less effective markers and spurious conclusions in practice.^43,66^ Here, AmpSeq markers with high *P*-values typically had extreme segregation distortion, indicating the segregation pattern of AmpSeq markers should be taken into consideration when choosing efficient markers.

### Advantage of AmpSeq genotyping platform

The most significant advantage of AmpSeq for MAS is the ability to harness the high-resolution GBS or other NGS techniques during marker development, which provides resiliency against rapid LD decay in species with high heterozygosity and diversity,^69^ while nearly eliminating issues of missing data and heterozygote under-calling common to GBS. An average density of 55, 218 and 133 kb per AmpSeq marker was obtained for flower sex, PM resistance and acylated anthocyanins, respectively. In grapevine, SSR and indel markers reported to predict flower sex^27^ have failed in progenies resulting from the cross between complex North American hybrids (BI Reisch and JJ Luby, personal communication), which may be due to the loss of linkage between the causal gene and markers. The situation has been similar for MAS of PM resistance. The closest pairs of *Ren2* SSR markers were 550-kb apart in the 12X.2 reference genome.^70–72^ Four amplicon markers were generated by the AmpSeq strategy within the 550-kb region, targeting a lower probability of recombination between causal gene and markers, and improved ability to detect recombinations near the locus.

AmpSeq gains efficiency via the NGS capacity to multiplex more markers per trait, more traits and more individuals. Our approach results in the genotyping of a haplotype block in one test for MAS decision making, which showed higher accuracy in cattle breeding^73,74^ and potential application in heterozygous crops such as cocoa^75^ and cotton,^76^ as well as for flowering time, cluster width and berry size in grapevine.^77^ While biallelic SNPs were traditionally criticized by limited polymorphisms per marker,^78^ the pooling of multiple SNP markers provides flexibility that overcomes this limitation.^79^ Further, multiple traits can be genotyped simultaneously for pyramiding, maximizing the application of MAS in contrast to phenotype screening. For example, anthocyanin pigments acylated to hydroxycinnamic acids are of interest to wine and juice producers because their absorbance behavior is not highly pH dependent, as compared with non-esterified anthocyanins in grapes which exist primarily in colorless forms over a juice pH range of 3–4 (ref. 80). Our results indicate that a hermaphrodite vine with increased resistance to PM and higher concentrations of berry acylated anthocyanins could be selected in a single AmpSeq run. While our current barcoding system is for 380 individuals (accommodating 4 blank negative controls), additional barcodes could be used for increased sample throughput.^39^

As mentioned above, marker transferability is another big concern in a breeding program with diverse germplasm spanning more than one species,^30^ and SNP microarrays for grapevine and apple are both reported to suffer from this constraint.^1,81,82^ Loss of polymorphism of SSR markers has also been reported when used in unrelated germplasm, as is the case for the marker *gwm261* for the locus *Reduced height 8* (*Rht8*) in wheat^83^ or *Ren4* in grapevine.^84^ This can result from sampling bias in the original study, since a distinct allele may not be amplified for the marker. The current work indicates that the amplicon markers are transferable in related families in grapevine. Among AmpSeq markers available in the current MAS package, dendrogram construction by genotype clustering could guide the choice of useful markers when transferring to new families without phenotypic data. In some, but not all cases, the markers most significantly associated with the trait clustered together (Figure 3), and in all cases markers not associated with the trait had poor correlations with the other markers. Still, because the amplicons are likely not genotyping the causal allele, re-training markers pools through validating, correcting and supplementing the current MAS package with markers developed in other breeding families by GBS or other genotyping platforms, following the same AmpSeq strategy, is recommended.

Finally, AmpSeq also provides advantages in terms of cost, time and ease of application. Actual cost to obtain AmpSeq data is comparable to single-locus SSRs and cheaper than most other multi-locus platforms. With the simple and straightforward PCR and library preparation protocol, the turn-around time from marker development through testing can be reduced to 1 month, which in our experience is quicker than other SNP platforms and SSRs. In addition, the data analysis pipeline can be automated to output results in a spreadsheet format, which may be attractive to some plant breeders, eliminating bioinformatic challenges typical of GBS data and removing the need for graphic interpretation typical of other genotyping platforms.

### A case of MAS implementation in grape breeding

The three traits selected for testing are economically important and representative targets for North American hybrid winegrape cultivar development: flower sex, PM resistance and acylated anthocyanins. Hybridization with wild species takes place to introgress positive adaptive traits including disease resistance, pest resistance and cold hardiness,^20,85–87^ but fruit quality and yield from the cultivated species *V. vinifera* are also critical. The families used for testing are five winegrape breeding families from Cornell University and the University of Minnesota, with diverse backgrounds including *V. vinifera*, *V. aestivalis*, *V. cinerea*, *V. labrusca*, *V. riparia* and *V. rupestris*. The New York and Minnesota breeding programs are connected by the historical use of ‘Seyval blanc’, which is a complex interspecific hybrid of *V. vinifera* (55% by pedigree) with wild species, and is a relatively cold hardy white wine cultivar, resistant to disease and the phylloxera aphid *Daktulosphaira vitifoliae* (Fitch, 1855).^85,88,89^ The current results demonstrate that the amplicon markers developed for all three traits are portable within the New York breeding program. Further, the flower sex amplicons are transferable between the New York and Minnesota programs. Since this study, we have implemented AmpSeq in breeding programs, and for each trait we have selected a subset of amplicons that appear to be robust and transferable. As an example, for 84 progeny of a *V. riparia* 37×Seyval blanc F_2_ family^90^ from the South Dakota State University breeding program, seven AmpSeq markers exceeded a 10^−8^ significance threshold for predicting female flower sex (data not shown)—an improvement over the results presented here, indicating the initial success of the MAS package implementation in an additional population.

### Customization of the AmpSeq strategy for other crops

In summary, the AmpSeq platform described here should provide several considerable advantages for breeders of other crop species due to its reliability, flexibility, high-throughput, cost-effectiveness, ease-of-automation and speed. This approach is among the first practical examples showing how SNP-based markers can be applied in a high-throughput screening, which may increase the application of GBS tags and leverage the massive power of NGS. This practicality may help fill the gap between genomic discoveries and breeding applications. With some customization, we propose that the AmpSeq strategy can be widely used by other crops.

## Figures and Tables

**Table 1:** QTL parameters for the development of AmpSeq markers

*Trait*	*Population*	*Haploblock start*	*Haploblock end*	*Anchor marker*	*QTL* R^*2*^ a	*1.8 LOD interval (left border)*	*1.8 LOD interval (right border)*	*Highest LOD*
Flower sex (M/H)	‘Chardonnay’×*V. cinerea* B9 (CC)	S2_4168128	S2_5507608	S2_5186869	99.5%	S2_4168128	S2_5186889	42.5
Flower sex (M/H)	‘Horizon’×*V. cinerea* B9 (HC)	S2_4178835	S2_5333625	S2_5186889	92.8%	S2_5186894	S2_5528872	39.8
Flower sex (M/H)	‘Horizon’×Illinois 547-1 (HI)	S2_4377285	S2_5333462	S2_5068641	97.0%	S2_4704546	S2_5068764	96.1
Flower sex (H/f)	MN1246×MN1264 (MN)	S2_4142601	S2_6967072	S2_5181254	82.1%	S2_4142601	S2_5715366	17.0
Powdery mildew in controlled environment	‘Horizon’×*V. cinerea* B9 (HC)	S14_25628594	S14_28890859	S14_27742679	12.7%	S14_26788064	S14_29638581	4.81
Acylated Anthocyanins	‘Horizon’×Illinois 547-1 (HI)	S3_14851567	S3_18229426	S3_17225376	54.0%	S3_8793603	S3_18757460	23.9

Abbreviations: QTL, quantitative trait loci.

a *R*^2^ (coefficient of determination): percentage of phenotypic variance explained by the QTL.

**Table 2:** AmpSeq marker-trait associations for flower sex

*Chr*	*Position*	*Family of AmpSeq design*	*Mean depth*	*CV*	*Missing individuals*	*Pooled (258)* a			*'Horizon'×Illinois 547-1 (87)*			*'Horizon'×V. cinerea B9 (82)*			*'Chardonnay'×V. cinerea B9 (89)*			*MN1246×MN1264 (47)*		
						*Flower sex (M/H)*			*Flower sex (M/H)*			*Flower sex (M/H)*			*Flower sex (M/H)*			*Flower sex (H/f)*		
						P*-value*	*marker R*^*2*^	*Minor allele frequency*	P*-value*	*marker R*^*2*^	*Minor allele frequency*	P*-value*	*marker R*^*2*^	*Minor allele frequency*	P*-value*	*marker R*^*2*^	*Minor allele frequency*	P*-value*	*marker R*^*2*^	*Minor allele frequency*
chr2	4245542	CC	248	1	0	1.64E−98	0.8238	0.27	1.92E−33	0.8204	0.31	2.32E−27	0.7714	0.24	2.99E−40	0.8696	0.24	NaN	0	0.08
chr2	4422069	CC	13	6	4	1.65E−13	0.1938	0.08	1.18E−03	0.1197	0.04	3.10E−05	0.1961	0.08	2.34E−06	0.2295	0.11	NaN	0	0
chr2	4428879	HI	109	42	1	7.18E−16	0.2256	0.10	4.57E−57	0.9517	0.30	NaN	0	0	NaN	0	0	NaN	0	0.08
chr2	4448335	HI	6	3	14	3.35E−06	0.0967	0.06	1.33E−06	0.2782	0.17	NaN	0	0	3.59E−01	0.0099	0.01	3.57E−01	0.0207	0.15
chr2	4457222	HI	248	2	0	1.66E−16	0.2336	0.10	2.96E−58	0.9531	0.29	NaN	0	0	NaN	0	0	4.54E−07	0.4852	0.48
chr2	4560461	HI	9	11	16	5.07E−146	0.9363	0.29	4.53E−49	0.9486	0.25	1.19E−32	0.8555	0.31	0.00E+00	1	0.29	1.29E−01	0.0505	0.01
chr2	4726521	HC	248	2	0	6.34E−17	0.2392	0.10	0.00E+00	1	0.29	NaN	0	0	NaN	0	0	5.23E−16	0.7711	0.25
chr2	4726692	HI	248	2	0	3.43E−173	0.9540	0.23	0.00E+00	1	0.20	7.68E−36	0.8595	0.24	0.00E+00	1	0.26	NaN	0	0
chr2	4798009	HI	96	49	1	8.30E−17	0.2385	0.10	0.00E+00	1	0.29	NaN	0	0	NaN	0	0	3.55E−01	0.0191	0.21
chr2	5005636	HI	248	1	0	5.52E−16	0.2264	0.10	4.58E−58	0.9526	0.30	NaN	0	0	NaN	0	0	5.23E−16	0.7711	0.25
chr2	5064267	MN	248	1	0	5.23E−16	0.7711	0.25	NaN^b^	0	0.49	NaN	0	0.48	3.23E−01	0.0112	0.24	5.23E−16	0.7711	0.25
chr2	5068641	HI	249	1	0	6.34E−17	0.2392	0.39	0.00E+00	1	0.20	NaN	0	0.48	NaN	0	0.50	NaN	0	0
chr2	5181254	MN	132	51	8	6.74E−14	0.2170	0.19	9.09E−41	0.9002	0.47	5.44E−01	0.0159	0.06	NaN	0	0	NaN	0	0.04
chr2	5207140	HI	7	5	25	3.47E−106	0.8712	0.26	1.76E−39	0.8985	0.21	2.59E−28	0.8414	0.29	1.54E−37	0.8671	0.27	NaN	0	0
chr2	5219708	HI	248	1	0	1.66E−16	0.2336	0.39	2.96E−58	0.9531	0.20	NaN	0	0.48	NaN	0	0.50	4.54E−07	0.4852	0.47
chr2	5219969	CC	248	1	0	3.38E−145	0.9239	0.23	2.96E−58	0.9531	0.20	7.68E−36	0.8595	0.24	9.85E−61	0.9559	0.26	NaN	0	0
chr2	5220041	HI	248	1	0	1.66E−16	0.2336	0.10	2.96E−58	0.9531	0.29	NaN	0	0	NaN	0	0	4.64E−04	0.2945	0.47
chr2	5233638	HI	181	68	107	1.20E−01	0.0257	0.06	5.79E−03	0.1554	0.02	6.22E−01	0.0373	0.18	2.34E−01	0.0196	0.03	NaN	0	0

Abbreviation: CC, ‘Chardonnay’×V. cinerea B9; CV, coefficient of variation of read depth across individuals; HC, 'Horizon’×V. cinerea B9; HI, ‘Horizon’×Illinois 547-1; MN, MN1246×MN1264; QTL, quantitative trait loci.

a Pooled includes all three families segregating M/H. Number of progeny analyzed is presented in parentheses.

b NaN means no association detected.

**Table 3:** AmpSeq marker-trait associations for *Ren2* powdery mildew resistance

*Chr*	*Position*	*Family of AmpSeq design*	*Mean depth*	*CV*	*Missing individuals*	*'Horizon'×V. cinerea B9 (78)* a			*'Horizon'×Illinois 547-1 (91)*		
						*Powdery mildew in controlled environment*			*Powdery mildew field rating*		
						P*-value*	*marker R*^*2*^	*Minor allele frequency*	P*-value*	*marker R*^*2*^	*Minor allele frequency*
chr14	26205721	HC	65	38	2	1.56E−02	0.1050	0.20	1.56E−05	0.1940	0.27
chr14	26954543	HC	207	40	0	9.23E−03	0.1174	0.27	2.24E−03	0.1309	0.47
chr14	27052970	HC	1	0	380	NaN^b^	0	0.00	NaN	0	0.00
chr14	27118778	HC	52	21	1	3.72E−03	0.1054	0.18	1.13E−07	0.2776	0.26
chr14	27263681	HC	232	5	2	2.42E−03	0.1148	0.19	1.45E−08	0.3101	0.26
chr14	27742679	HC	244	2	1	1.34E−03	0.1274	0.18	2.14E−08	0.3041	0.27
chr14	28408093	HC	248	2	0	6.12E−04	0.1440	0.19	3.82E−06	0.2165	0.27
chr14	28415565	HC	248	2	0	6.12E−04	0.1440	0.19	9.53E−06	0.2007	0.26
chr14	28448817	HC	51	48	42	3.78E−03	0.1399	0.34	1.05E−02	0.1205	0.37
chr14	28686983	HC	248	2	0	8.90E−03	0.0866	0.20	8.29E−06	0.2031	0.27
chr14	28791319	HC	50	38	2	1.32E−02	0.1089	0.25	6.44E−05	0.2010	0.34
chr14	28824733	HC	101	49	2	1.32E−02	0.0782	0.20	4.53E−05	0.1749	0.28

Abbreviations: CV, coefficient of variation of read depth across individuals; HC, 'Horizon’×V. cinerea B9; QTL, quantitative trait loci.

a Number of progeny analyzed is presented in parentheses.

b NaN means no association detected.

**Table 4:** AmpSeq marker-trait associations for acylated anthocyanins

*Chr*	*Position*	*Family of AmpSeq design*	*Mean depth*	*CV*	*Missing individuals*	*Pooled (182)* a			*'Horizon'×Illinois 547-1 (82)*			*V. rupestris B38×'Horizon' (100)*		
						*Acylated anthocyanins*			*Acylated anthocyanins*			*Acylated anthocyanins*		
						P*-value*	*marker R*^*2*^	*Minor allele frequency*	P*-value*	*marker R*^*2*^	*Minor allele frequency*	P*-value*	*marker R*^*2*^	*Minor allele frequency*
chr3	14851567	HI	250	0	0	1.07E−16	0.3370	0.33	7.36E−06	0.2586	0.42	4.90E−13	0.4147	0.25
chr3	15171738	HI	247	2	0	2.05E−17	0.3491	0.28	7.31E−06	0.2587	0.30	5.33E−13	0.4137	0.25
chr3	15828002	HI	250	0	127	1.76E−10	0.3102	0.47	8.82E−03	0.1724	0.44	8.35E−09	0.4214	0.50
chr3	16007996	HI	245	3	0	1.26E−16	0.3357	0.26	5.35E−06	0.2646	0.26	4.10E−12	0.3892	0.26
chr3	16008017	HI	244	3	0	1.26E−16	0.3357	0.26	5.35E−06	0.2646	0.26	4.10E−12	0.3892	0.26
chr3	16162132	HI	250	0	67	2.70E−03	0.0763	0.31	2.73E−04	0.2502	0.43	3.78E−02	0.0710	0.21
chr3	16163517	HI	249	1	0	3.64E−17	0.3265	0.24	1.15E−06	0.2574	0.22	4.10E−12	0.3892	0.26
chr3	16246446	HI	241	5	0	5.22E−03	0.0425	0.05	6.13E−02	0.0431	0.06	5.29E−02	0.0377	0.05
chr3	16375440	HI	249	1	0	3.64E−17	0.3265	0.24	1.15E−06	0.2574	0.22	4.10E−12	0.3892	0.26
chr3	16402674	HI	113	39	1	1.61E−06	0.1385	0.42	6.88E−06	0.2599	0.35	1.35E−04	0.1678	0.50
chr3	16402706	HI	14	6	2	2.15E−15	0.3143	0.33	7.62E−06	0.2580	0.29	1.59E−10	0.3720	0.37
chr3	16473116	HI	249	1	0	1.67E−16	0.3336	0.43	2.06E−05	0.2391	0.36	1.86E−11	0.3992	0.49
chr3	16516083	HI	4	4	200	1.80E−05	0.2417	0.35	8.50E−04	0.2507	0.46	NaN	0	0.01
chr3	16520617	HI	249	1	0	8.04E−17	0.3390	0.24	1.15E−06	0.2574	0.23	4.78E−12	0.4158	0.26
chr3	16523313	HI	73	53	3	1.85E−16	0.3329	0.30	1.15E−06	0.2574	0.22	6.12E−11	0.3842	0.38
chr3	16774466	HI	249	1	0	1.06E−17	0.3356	0.26	1.26E−06	0.2557	0.29	5.02E−13	0.4145	0.24
chr3	16873018	HI	248	2	0	NaN^b^	0	0.00	NaN	0	0.01	NaN	0	0.00
chr3	16873164	HI	88	46	0	1.06E−17	0.3356	0.26	1.26E−06	0.2557	0.28	5.02E−13	0.4145	0.24
chr3	17225240	HI	20	27	2	9.37E−14	0.2861	0.22	6.59E−05	0.2163	0.18	2.53E−10	0.3393	0.27
chr3	17225412	HI	149	0	31	8.45E−03	0.0413	0.06	2.17E−02	0.0720	0.06	1.06E−01	0.0281	0.06
chr3	17225585	HI	210	14	0	2.28E−17	0.3483	0.49	6.66E−07	0.3024	0.45	1.96E−11	0.3985	0.48
chr3	17774559	HI	62	151	124	1.97E−02	0.0649	0.31	1.08E−02	0.1319	0.22	4.35E−01	0.0328	0.44

Abbreviations: CV, coefficient of variation of read depth across individuals; HI, ‘Horizon’×Illinois 547-1; QTL, quantitative trait loci.

a Pooled includes all two families. Number of progeny analyzed is presented in parentheses.

b NaN means no association detected.
